# Inhibition of hepatitis B viral entry by nucleic acid polymers in HepaRG cells and primary human hepatocytes

**DOI:** 10.1371/journal.pone.0179697

**Published:** 2017-06-21

**Authors:** Clément Guillot, Nora Martel, Françoise Berby, Isabelle Bordes, Olivier Hantz, Matthieu Blanchet, Camille Sureau, Andrew Vaillant, Isabelle Chemin

**Affiliations:** 1 Centre de Recherche en Cancérologie de Lyon INSERM U1052, CNRS UMR5286, Université de Lyon, Lyon, France; 2 Replicor Inc. Montréal, Canada; 3 Molecular Virology Laboratory, Institut National de la Transfusion Sanguine (INTS), CNRS INSERM U1134, Paris, France; Defence Research Laboratory, INDIA

## Abstract

Hepatitis B virus (HBV) infection remains a major public health concern worldwide with 240 million individuals chronically infected and at risk of developing cirrhosis and hepatocellular carcinoma. Current treatments rarely cure chronic hepatitis B infection, highlighting the need for new anti-HBV drugs. Nucleic acid polymers (NAPs) are phosphorothioated oligonucleotides that have demonstrated a great potential to inhibit infection with several viruses. In chronically infected human patients, NAPs administration lead to a decline of blood HBsAg and HBV DNA and to HBsAg seroconversion, the expected signs of functional cure. NAPs have also been shown to prevent infection of duck hepatocytes with the Avihepadnavirus duck hepatitis B virus (DHBV) and to exert an antiviral activity against established DHBV infection *in vitro* and *in vivo*.

In this study, we investigated the specific anti-HBV antiviral activity of NAPs in the HepaRG human hepatoma cell line and primary cultures of human hepatocytes. NAPs with different chemical features (phosphorothioation, 2’O-methyl ribose, 5-methylcytidine) were assessed for antiviral activity when provided at the time of HBV inoculation or post-inoculation. NAPs dose-dependently inhibited HBV entry in a phosphorothioation-dependent, sequence-independent and size-dependent manner. This inhibition of HBV entry by NAPs was impaired by 2’O-methyl ribose modification. NAP treatment after viral inoculation did not elicit any antiviral activity.

## Introduction

Hepatitis B virus (HBV) infection remains a global health problem. An estimated 2 billion individuals have been infected, representing approximately 30% of world’s population, and more than 240 million remain chronically infected [1] and at risk of developing cirrhosis and hepatocellular carcinoma. More than 780,000 patients die each year due to hepatitis B complications, ranking this infectious disease as the tenth leading cause of death in the 2010 Global Burden of Disease study [1,2]. HBV infection is unique in that infected cells produce and secrete copious amounts of non-infectious subviral particles covered with the viral surface glycoprotein (HBsAg), which constitute the bulk of circulating viral particles [3]. The abundant circulating HBsAg is thought to exhaust the HBV specific B and T cell responses, interfere with signaling in adaptive and innate immune function and to contribute to viral persistence [4,5].

Current antiviral therapies can lower circulating HBV DNA to undetectable levels but suppress serum HBsAg only very infrequently [6–9], which is the likely reason for the inability of these treatments to restore functional control of HBV infection.

Nucleic acid polymers (NAPs) are oligonucleotide-based compounds that display broad spectrum antiviral activity in enveloped viruses [10]. The antiviral activity of NAPs against several enveloped viruses is strictly dependent on amphipathicity conferred by phosphorothioation [11,12]. This antiviral activity was additionally shown to be sequence independent but length dependent. These features of NAPs are thought to confer interaction with non-complexed amphipathic α-helices present on surface glycoproteins of various enveloped viruses [11,12] such as HIV-1, LCMV, HSV-2, CMV and HCV [11–16]. Additionally, phosphorothioated oligonucleotides as class of compounds, regardless of sequence, are rapidly cleared from the blood concomitant with their accumulation in the liver, kidney, lung and spleen, making NAPs ideal for the treatment of viral infections in these organs [10] (Ingo Roehl, personal communication).

Importantly, NAPs have been shown to clear surface antigen in the DHBV pre-clinical model and in proof of concept clinical studies with patients chronically infected with HBV [17–19]. *In vitro*, NAPs have been shown to exert an anti-DHBV activity in primary duck hepatocyte cultures when added co- or post-inoculation. However, in the duck model, the entry-inhibitory effects of NAP *in vitro* do not appear to be mandatory to the antiviral effect *in vivo* [20].

To gain more insight on NAP antiviral activity against HBV, we treated HepaRG cells or primary human hepatocytes (PHH) at the time of HBV inoculation, or post-inoculation, and subsequently measured several markers of HBV infection. We demonstrate that NAPs exert a strong antiviral activity at viral entry at non-toxic concentrations. Optimal NAP antiviral activity requires phosphorothioation, and is size-dependent and sequence-independent. Additionally, the inhibition of viral entry by NAPs is impaired by 2’O-methylation.

## Materials and methods

### Cell culture

HepG2 2.2.15 [21] were maintained in DMEM medium with 10% fetal bovine serum and 1% penicillin/streptomycin, 1% Glutamine, 1% non-essential amino acids, 1% Sodium Pyruvate and 380 μg/ml G418. Primary human hepatocytes were isolated from surgical liver specimens obtained during partial hepatectomy. Informed consent was obtained from each patient, and the procedure was approved by the local Ethics Committee CPP Sud-Est IV (Agreements of the French Ministry of Education and Research n° AC-2013-1871 and n° DC-2013-1870, ISO certification n° NFS 96 900). HepaRG cells [22] were maintained in William’s E medium with 10% Fetal Clone II fetal bovine serum, 1% penicillin/streptomycin, 0.4% Gentamicin, 1% Glutamine supplemented with 7 x 10^-5^M hydrocortisone hemisuccinate, 5 μg/ml insulin. Prior to inoculation, confluent HepaRG cells were differentiated for two weeks with the culture medium described above and for a minimum of two additional weeks using the same culture medium supplemented with 1.8% DMSO. For PHH infection assays, cells were grown in HepaRG culture medium without FBS for one day and then HepaRG culture medium with 5% Fetal Clone II FBS and 2.2% DMSO.

### HBV production

For the production of HBV particles, HepG2 2.2.15 cells were grown as described above [21]. They were maintained in a mixed medium of HepG2 2.2.15 medium and HepaRG medium at a ratio of 1:1 supplemented with 2% DMSO. HBV particles were concentrated from the clarified supernatant by overnight precipitation with 5% PEG 8000 and centrifugation at 4°C for 4 h at 3900 rpm. Pellets were resuspended in cold 1X PBS and ultracentrifuged on a 5–15% sucrose cushion at 27,000 rpm during 16h at 4°C. The pellet was resuspended in Opti-MEM medium supplemented with 1% DMSO at a concentration of 10^10^ genome equivalent (ge) per ml.

### HBV infection assay

Prior to inoculation, differentiated HepaRG cells were treated with 1X EGTA (Sigma Aldrich) for 1h at 37°C, 5% CO_2_ and were inoculated with 500 ge per cell in complete medium containing, 1.8% DMSO, 4% PEG 8000, 10-fold the normal amount of hemisuccinate hydrocortisone and 1X EGTA. Cells were incubated 1h at room temperature and 20h at 37°C and 5% CO_2_. Cells were washed five times in complete medium containing 1.8% DMSO. PHH were inoculated using the same procedure in the absence of EGTA treatment. NAPs were resuspended in PBS and added to cells medium at the indicated time and concentrations.

### HBsAg and HBeAg quantification

Secreted HBsAg was measured quantitatively using the ElecsysHBsAg assay (ROCHE diagnostics). Hepatitis B virus e antigen (HBeAg) was measured using the HBEAG Chemiluminescence Immunoassay kit (Autobio Diagnostics Co., Ltd).

### Quantitative real-time PCR

Absolute and relative quantification were performed using complete thermocycling parameters previously described [23] using the iQ SYBR Green Supermix kit (Bio-Rad). All samples were assessed in duplicates. HBV DNA from HBV particles production was extracted using the High Pure Viral Nucleic Acid Kit (ROCHE diagnostics). Absolute quantification was performed as previously described [23] using a standard curve by serial dilution of a cloned HBV (ayw, genotype D in pTriEx vector) plasmid and the following primers HBV FW: 5’-GCTGACGCAACCCCCACT-3’ and HBV R: 5’-AGGAGTTCCGCAGTATGG-3’ (Amplicon length: 98 bp). For HBV RNA analysis, total RNA was extracted with the MasterPure RNA Purification Kit (Epicentre) following manufacturer’s instructions. RNA was reverse-transcribed using the Superscript VILO cDNA Synthesis Kit (Thermo Fisher Scientific) following manufacturer’s instructions. Quantification was performed using the HBV FW and HBV R primers. Values were normalized to the beta-glucuronidase (GUSB) expression levels (GUSB FW: 5’–CGTGGTTGGAGAGCTCATTTGGAA-3’; GUSB R: 5’-ATTCCCCAGCACTCTCGTCGGT-3’; Amplicon length: 73 bp) using the ΔΔCt method as previously described [24].

### Cytotoxicity assay

HepaRG cells were differentiated as described above and were infected (+ HBV condition) or not (- HBV condition) with HBV inoculum. At day 1 post-infection, cells were trypsinized and seeded in 96-well plates at a density of 1.5 x 10^5^cells/well. Cells were treated with NAPs at day 2, 4, and 6 post-infection. At day 8, toxicity was assessed using the neutral red assay [25].

### Statistical analysis

Experiments were reproduced independently two to four times with internal biological duplicates, except for the HBV RNA data obtained in PHH with REP 2139 post-viral inoculation treatment which has been done once. All data were expressed as means ± standard deviation. Statistical analysis was conducted with R software using an ordinary one-way ANOVA with random effect for comparison to non-treated sample, or unpaired, 2-tailed t-tests for comparison of specific samples using GraphPadPrism6 software;*, p < 0.05; **, p < 0.01; ***, p < 0.001; ****, p < 0.0001.

## Results

NAPs with different chemical features are listed in Table 1. Briefly, they are poly-C, poly-AC repeat-based or degenerate oligonucleotides containing, or not, the following modifications: phosphorothioation (PS) increasing stability and amphipathicity, 2’O-methyl ribose modification (2’O-Me) increasing stability and reducing TLR reactivity and methylation of cytosine bases (5-MeC) reducing TLR reactivity. REP 2138, REP 2172 and REP 2147 were used as non-PS non-amphipathic (polyanionic) control NAPs. Phosphorothioated poly-AC NAPs of different lengths (60, 40, 30, 20, and 10 nucleotides) were also used.

**Table 1:** Chemical characteristics of the different NAPs used in this study.

Name	Sequence (5’– 3’)	Modification			Activity
		PS^b^	2'O-Me^c^	5-MeC^d^	
Chemistry and Sequence Modifications (all 40mer)					
REP 2006	NNNNNNNNNNNNNNNNNNNNNNNNNNNNNNNNNNNNNNNN^a^	+			+++
REP 2107	NNNNNNNNNNNNNNNNNNNNNNNNNNNNNNNNNNNNNNNN	+	+		+
REP 2055	ACACACACACACACACACACACACACACACACACACACAC	+			+++
REP 2139	ACACACACACACACACACACACACACACACACACACACAC	+	+	+	+
REP 2165	ACACACACACACACACACACACACACACACACACACACAC	+	+^e^	+	+
REP 2172	ACACACACACACACACACACACACACACACACACACACAC		+		-
REP 2147	ACACACACACACACACACACACACACACACACACACACAC		+	+	-
REP 2031	CCCCCCCCCCCCCCCCCCCCCCCCCCCCCCCCCCCCCCCC	+			+++
REP 2138	CCCCCCCCCCCCCCCCCCCCCCCCCCCCCCCCCCCCCCCC		+		-
Size variations					
REP 2149	ACACACACACACACACACACACACACACACACACACACACACACACACACACACACACAC (60mer)	+			+++
REP 2055	ACACACACACACACACACACACACACACACACACACACAC (40mer)	+			+++
REP 2150	ACACACACACACACACACACACACACACAC (30mer)	+			++
REP 2151	ACACACACACACACACACAC (20mer)	+			+
REP 2152	ACACACACAC (10mer)	+			-

^a^N = random mixture of A, G, T and C at each nucleotide position

^b^PS = phosphorothioation of phosphodiester linkage (increased amphipathicity and stability)

^c^2’O-Me = O-linked methylation at 2’ position in ribose (increased stability and reduced TLR reactivity)

^d^5-MeC = methylation of 5’ position in cytidine base (reduced TLR reactivity)

^e^Positions 11, 21 and 31 have 2’OH ribose.

### NAPs suppress HBV infection of HepaRG cells and PHH

**Fig 1:**
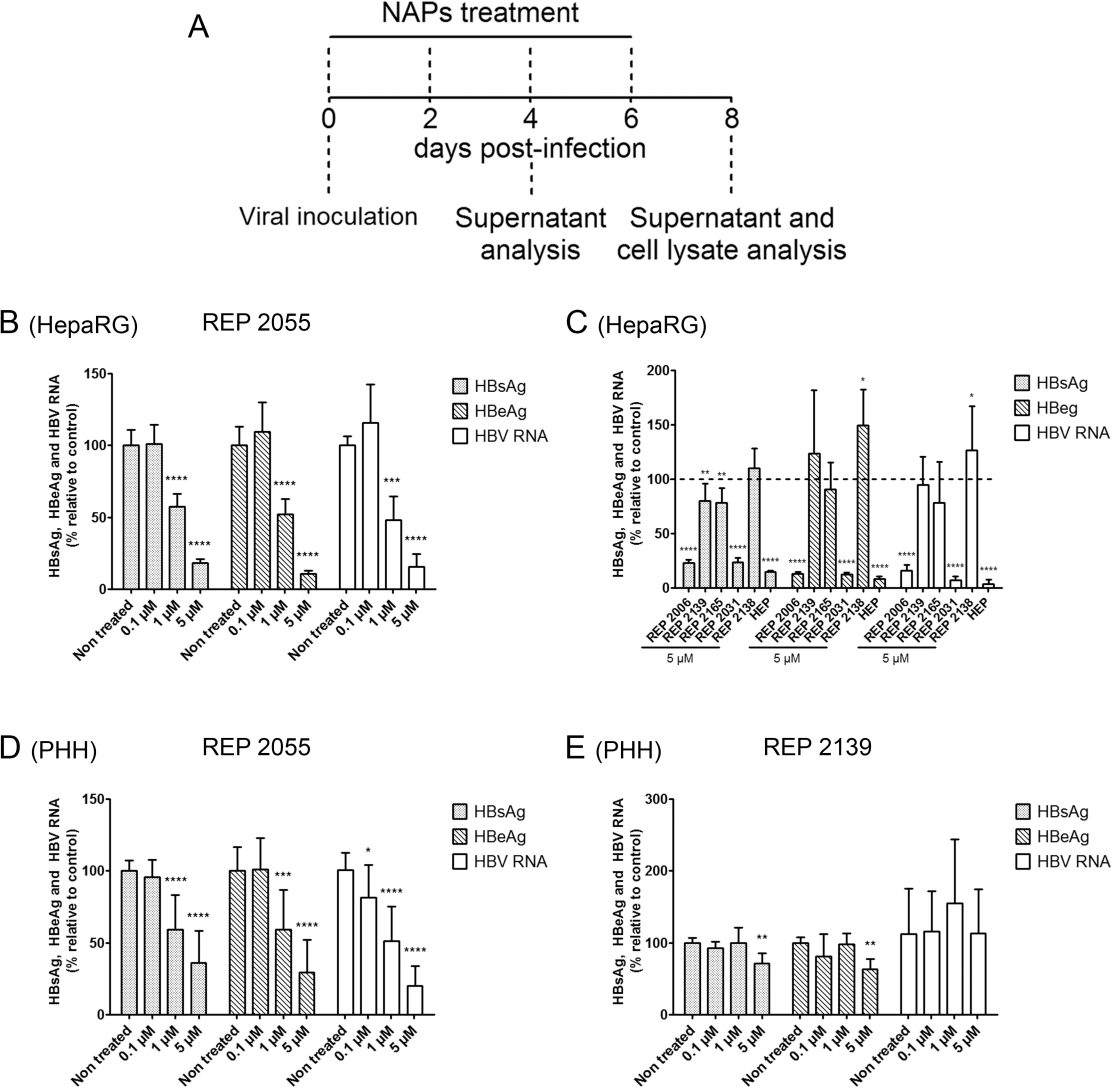
Effect of multiple nucleic acid polymers treatments starting at the time of viral inoculation on HBV replication in HepaRG cells and primary human hepatocytes. (A) Treatments procedure: HBV infected HepaRG cells and primary human hepatocytes were treated every two days starting at the time of inoculation. Supernatants and cell lysates were harvested for extracellular HBsAg, HBeAg and intracellular total HBV RNA analysis at day 4 (data not shown) and day 8 post-inoculation. The antiviral effect of (B) REP 2055 at 0.1 μM, 1 μM and 5 μM final concentrations and (C) REP 2006, REP 2139, REP 2165, REP 2031 and REP 2138 at 5 μM final concentration was assessed on differentiated HepaRG cells at day 8 post-inoculation by measuring secreted HBsAg, HBeAg and total HBV RNA. The antiviral effect of (D) REP 2055 and (E) REP 2139 at 0.1 μM, 1 μM and 5 μM final concentrations was assessed on primary human hepatocytes at day 8 post-inoculation by measuring secreted HBsAg, HBeAg and total HBV RNA. The solvent of NAP compounds was used as a non-treated condition. Heparin (HEP) was used as a positive control for entry inhibition at a concentration of 300 μg/ml. All data, expressed as means ± standard deviation, were independently reproduced three to four times, except for the effect of REP 2055 and REP 2139 on HBV RNA in HepaRG which was reproduced two times. Statistical analysis was conducted with R software using an ordinary one-way ANOVA with random effect for comparison to non-treated sample; *, p < 0.05; **, p < 0.01; ***, p < 0.001; ****, p < 0.0001.

Cells were treated with non-toxic concentrations of NAPs (S1 Fig) provided at the time of inoculation and for 8 days post-inoculation (Fig 1A). At day 8 post-inoculation, supernatants were harvested and tested for the presense of HBsAg and HBeAg, and cell lysates were tested for the presence of HBV RNA. A dose-dependent decrease of secreted HBsAg and HBeAg and of total intracellular HBV RNA was observed in both HepaRG cells and PHH cultures upon treatment with the AC-repeat based REP 2055 (Fig 1B and 1D). A decrease of HBsAg, HBeAg and HBV RNA was also observed upon treatment of HepaRG cells with 5 μM REP 2006 (a degenerate 40-mer), or REP 2031 (a poly-C 40-mer) (Fig 1C). REP 2139 and REP 2165 (REP 2055 derivatives with 2’O-Me and 5-MeC) showed a weak or no effect in HepaRG cells (Fig 1C). REP 2139 activity was also reduced in PHH as compared to REP 2055 (Fig 1D and 1E). The non-phosphorothioated control compound REP 2138 did not show any antiviral activity (Fig 1C). Heparin, which inhibits HBV attachment to heparan sulfate proteoglycan (HSPG) [26] was used as a positive control in HepaRG cells for entry inhibition (Fig 1C).

### NAPs have no effect on HBV infection when provided post-inoculation

**Fig 2:**
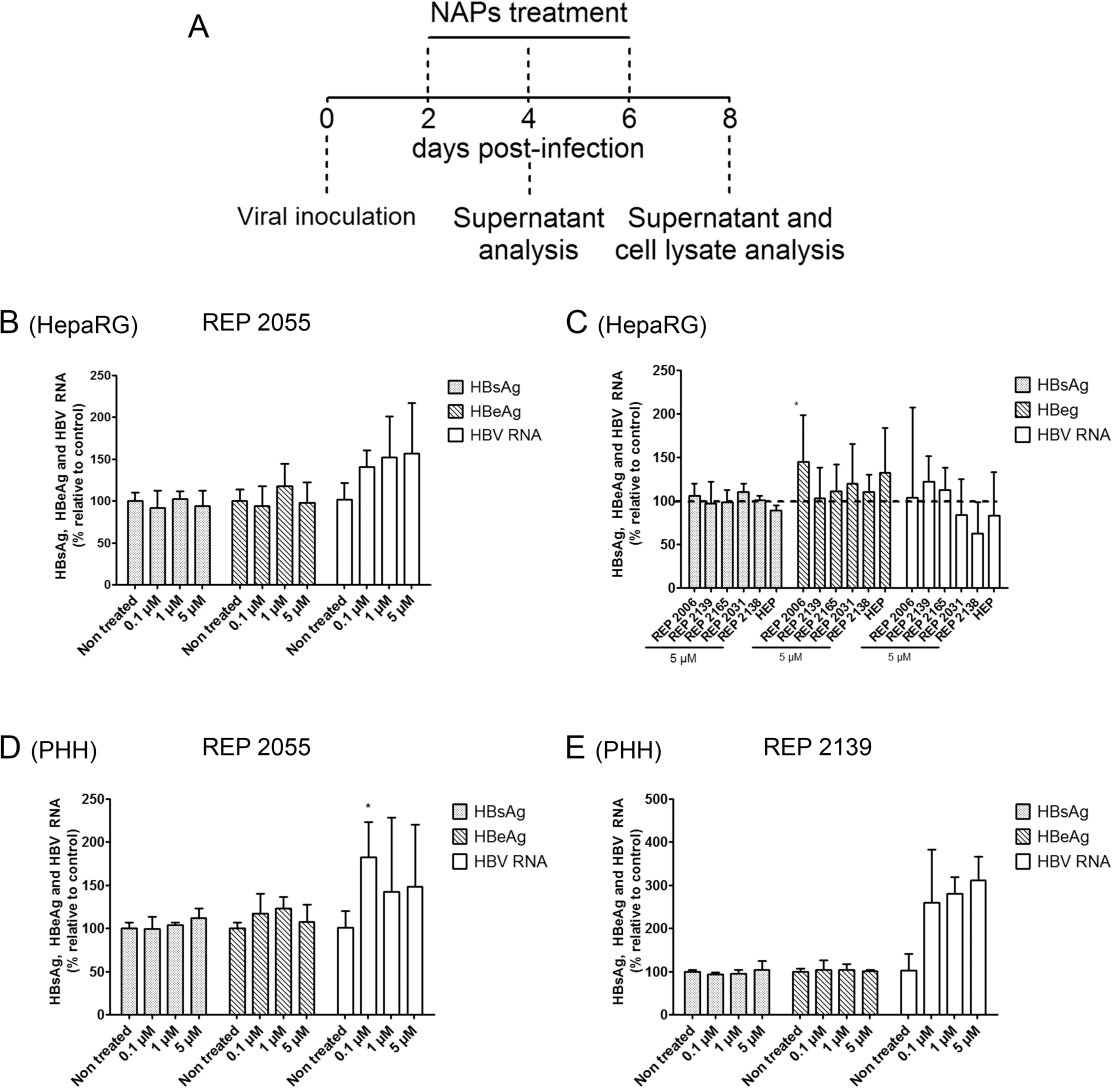
Effect of post-viral inoculation treatment with nucleic acid polymers on HBV replication in HepaRG and primary human hepatocytes. (A) Treatments procedure: HBV infected cells were treated every two days starting two days post-inoculation with (B) REP 2055 at 0.1 μM, 1 μM and 5 μM final concentrations,(C) REP 2006, REP 2139, REP 2165, REP 2031 and REP 2138 at 5 μM final concentration in differentiated HepaRG cells and (D) REP 2055 and (E) REP 2139 at 0.1 μM, 1 μM and 5 μM final concentrations in primary human hepatocytes at day 8 post-inoculation by measuring secreted HBsAg, HBeAg and total HBV RNA (See legend to Fig 1. for experimental details). The solvent of NAP compounds was used as a non-treated condition. Heparin (HEP) was used as a positive control for entry inhibition at a concentration of 300 μg/ml. All data, expressed as means ± standard deviation, were independently reproduced two to four times, except for the effect of REP 2139 on HBV RNA which was reproduced one time in PHH. Statistical analysis was conducted with R software using an ordinary one-way ANOVA with random effect for comparison to non-treated sample; *, p < 0.05; **, p < 0.01; ***, p < 0.001; ****, p < 0.0001.

HepaRG cells and PHH were treated with NAPs every two days starting at day 2 post-inoculation (Fig 2A). In this context of addition of NAPs without transfection, no effect on viral markers could be observed even with the most potent NAPs (Fig 2). As expected, treatment with heparin after the establishment of infection failed to exert any antiviral effect (Fig 2C).

### NAPs suppress HBV infection of HepaRG cells and PHH at viral entry

**Fig 3:**
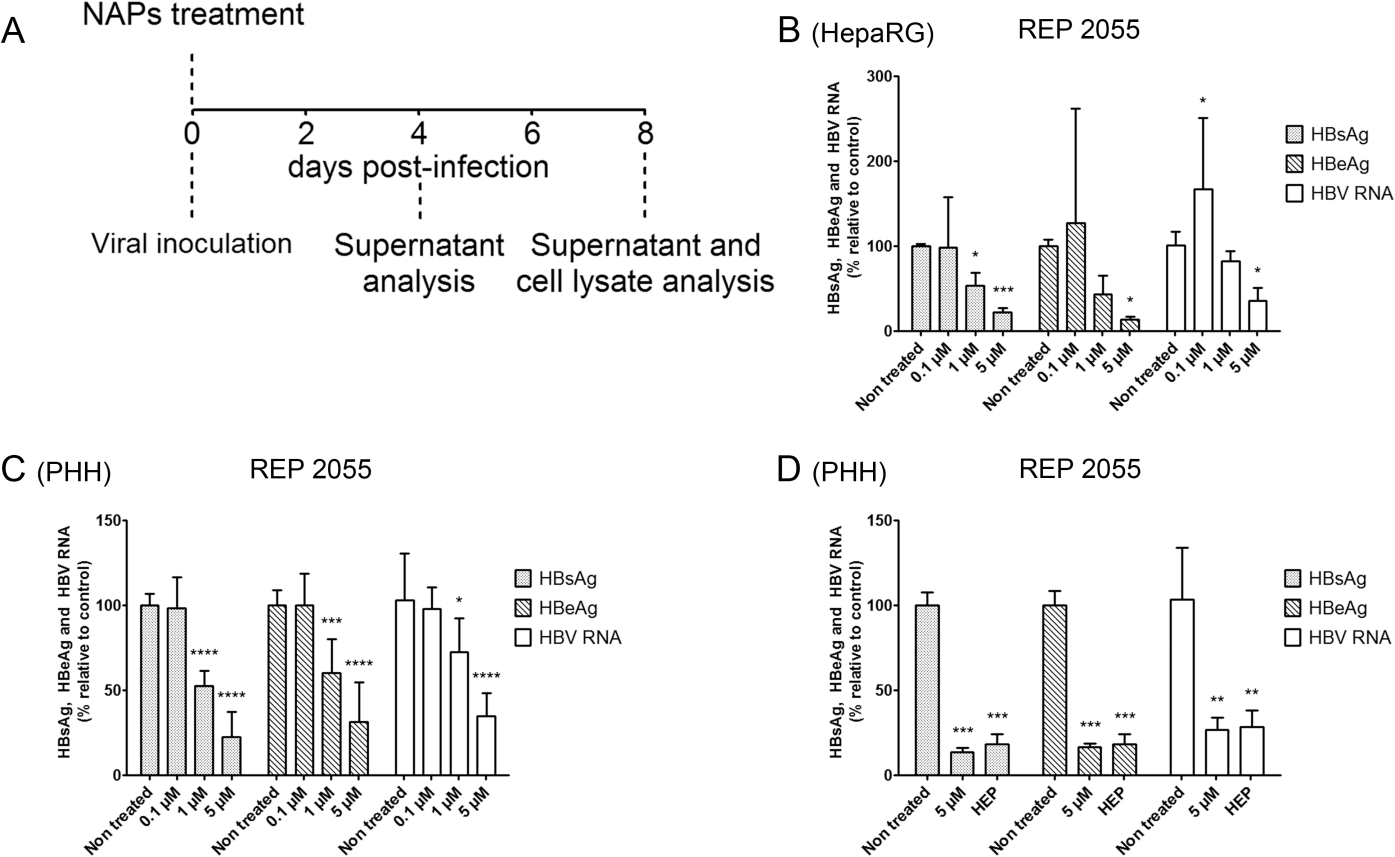
Effect of a single nucleic acid polymers treatment at the time of viral inoculation on HBV replication in HepaRG cells and primary human hepatocytes. (A) Treatments procedure: HBV infected cells were treated at the time of inoculation for the duration of inoculation with REP 2055 at 0.1 μM, 1 μM and 5 μM final concentrations in (B) differentiated HepaRG cells and (C) primary human hepatocytes at day 8 post-inoculation by measuring secreted HBsAg, HBeAg and total HBV RNA. (D) In primary human hepatocytes, in a side by side experiment with 5 μM REP 2055, heparin (HEP) was used as a positive control for entry inhibition at a concentration of 300 μg/ml (See legend to Fig 1. for experimental details). All data originate from three independent experiments except in (D) where two independent experiments have been performed. Results are expressed as means ± standard deviation. Statistical analysis was conducted with R software using an (B, C) ordinary one-way ANOVA with random effect for comparison to non-treated sample and (D) unpaired, 2-tailed t-tests for comparison of specific samples using GraphPadPrism6 software; *, p < 0.05; **, p < 0.01; ***, p < 0.001; ****, p < 0.0001.

To gain insight into the NAPs antiviral activity at HBV entry, HepaRG cells and PHH cultures were exposed to REP 2055 during viral inoculation only (Fig 3A). An antiviral activity similar to that shown in Fig 1 was observed (Fig 3B, 3C and 3D). Heparin was used as a positive control (Fig 3D). These results, along with the observation of a lack of antiviral effect upon addition of NAPs post-inoculation, demonstrate that NAPs are active at viral entry.

### 2’O-Me modification decreases the antiviral activity of NAPs at HBV viral entry

**Fig 4:**
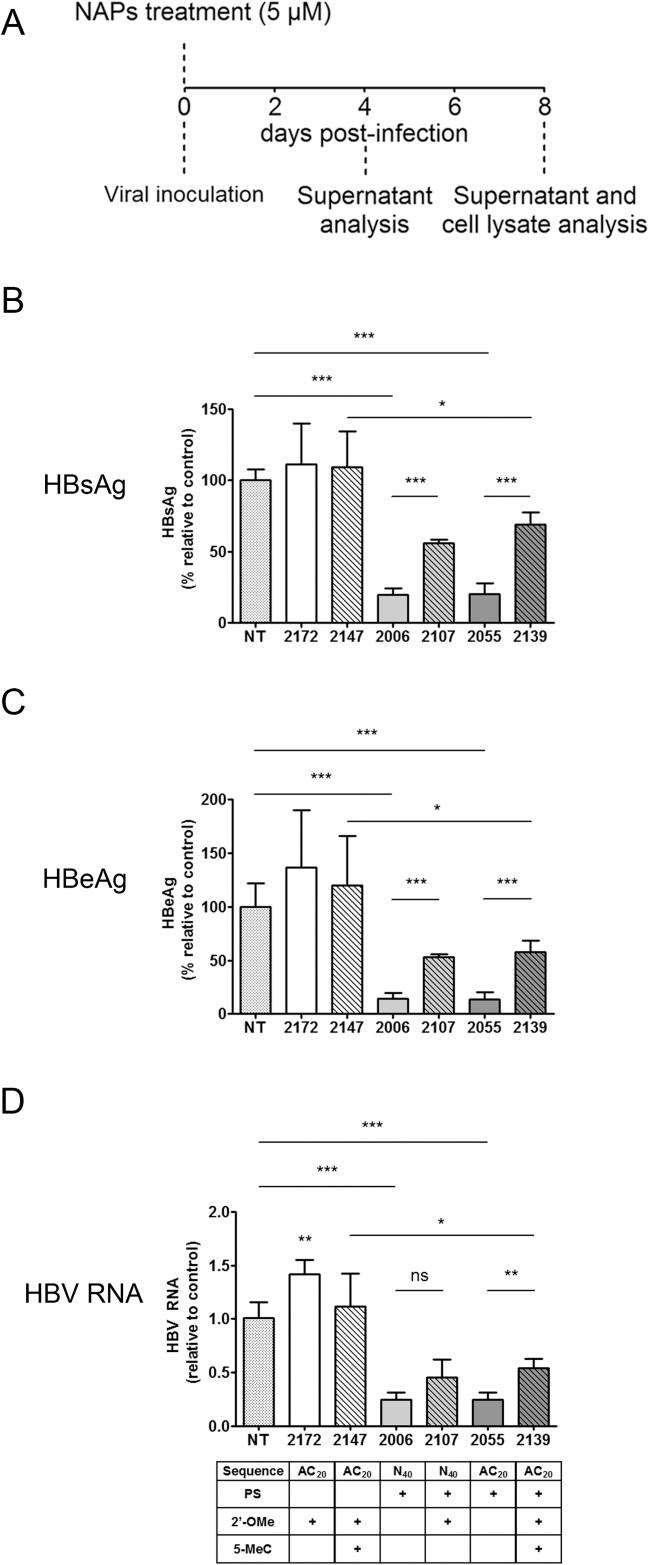
Relationship between NAPs sequence, amphipathicity, chemical modification and anti-HBV antiviral activity. (A) Treatments procedure: HBV infected HepaRG cells were treated at the time of inoculation for the duration of inoculation. Supernatants and cell lysates were harvested for extracellular HBsAg, HBeAg and intracellular total HBV RNA analysis at day 4 (data not shown) and day 8 post-inoculation. (B) To determine the effect of different NAPs chemical modifications on antiviral activity, HepaRG cells were treated once at the time of viral inoculation with REP 2172, REP 2147, REP 2006, REP 2107, REP 2055 and REP 2139 NAP compounds containing or not PS (phosphorothioation), 2’-OMe (2’O-methyl ribose) and 5-MeC (5-methylcytidine) at a 5 μM concentration and secreted HBsAg, HBeAg and total HBV RNA were measured at day 8 post-inoculation. The solvent of NAP compounds was used as a non-treated condition. All data originating from two independent experiments are expressed as means ± standard deviation. Statistical analysis was conducted with unpaired, 2-tailed t-tests, for comparison of specific samples; *, p < 0.05; **, p < 0.01; ***, p < 0.001; ****, p < 0.0001, ns; non-significant.

REP 2139 is identical in sequence to REP 2055 but is 2’O-Methylated. While exhibiting a similar antiviral activity in proof of concept clinical trials, the *in vitro* ability of REP 2139 (2’O-Methylated) to impair viral entry was significantly reduced as compared to REP 2055 (Figs 1 and 4). This result obtained with poly-AC sequences was confirmed using NAPs with degenerate sequences with or without 2’O-Me (REP 2107 and REP 2006, respectively) (Fig 4).

### Phosphorothioation of NAPs is required for activity

The use of a control NAP devoid of both phosphorothioation and 2’O-Me modification is technically impossible due to very poor molecular stability [12]. However, the requirement of phosphorothioation for proper HBV-antiviral activity is demonstrated by the fact that phophorothioated NAPs without 2’O-Me (*e*.*g*. poly-AC repeat-based REP 2055) show potent activity, while a non-phosphothioated NAP with 2’O-Me (*e*.*g*. poly-AC repeat-based REP 2172) which is equally stable, was inactive (Fig 4). Moreover, a stronger activity was observed with REP 2139 as compared to its non-phosphorothioated counterpart REP 2147 (Fig 4).

### Entry inhibition activity of NAPs is sequence-independent

The antiviral effect of NAPs on enveloped viruses has been shown to be sequence independent [11,12]. Here, cells treatment with 40-mer NAPs consisting of either a degenerate sequence (REP 2006), an AC repeat-based sequence (REP 2055), or a poly-C sequence (REP 2031) resulted in comparable inhibition of viral entry (Figs 1C and 4) leading to approximately 80% reduction of HBsAg and HBeAg secretion and intracellular HBV RNA. Interestingly, the presence of CpG motifs in the degenerate REP 2006 did not confer a stronger antiviral activity as compared to NAPs of defined sequences (Figs 1C and 4).

### Entry inhibition activity of NAPs is size-dependent

**Fig 5:**
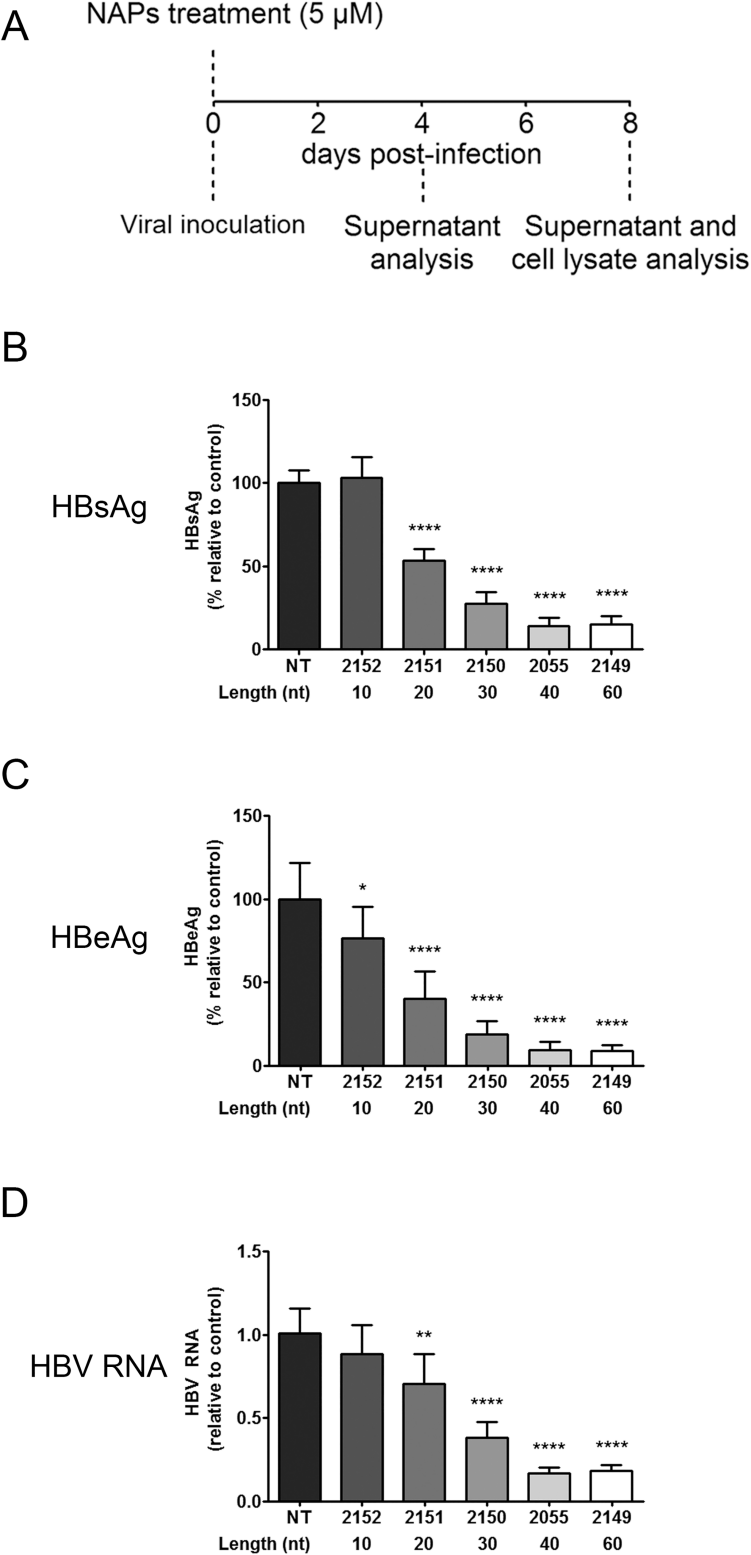
Relationship between NAPs size and anti-HBV antiviral activity. (A) Treatments procedure: HBV infected HepaRG cells were treated at the time of inoculation for the duration of inoculation. Supernatants and cell lysates were harvested for extracellular HBsAg, HBeAg and intracellular total HBV RNA analysis at day 4 (data not shown) and day 8 post-inoculation. (B) The effect of NAPs size on antiviral activity was assessed by measuring the effect of REP 2055 analogs of different length from 60 to 10 nucleotides length on secreted HBsAg, HBeAg and total HBV RNA in HepaRG cells treated once at the time of viral inoculation at a 5 μM NAPs concentration. The solvent of NAP compounds was used as a non-treated condition. All data originating from two independent experiments are expressed as means ± standard deviation. Statistical analysis was conducted with R software using an ordinary one-way ANOVA with random effect for comparison to non-treated sample; *, p < 0.05; **, p < 0.01; ***, p < 0.001; ****, p < 0.0001.

NAPs antiviral activity towards enveloped viruses has been shown to be size dependent [11,12]. To confirm that NAP size is an important feature for anti-HBV antiviral activity, we measured the activity of REP 2055 derivatives of different lengths (60, 40, 30, 20, and 10 nucleotides) in HepaRG cells in a coinoculation assay (Fig 5). Results show that the 60- and 40-mer exerted a strong antiviral activity with a maximum effect for the 40-mer REP 2055. The antiviral activity decreased gradually with length and was lost for 10-mer REP 2152 (Fig 5). Consistent with previous studies with other viruses [11–14,16], a length of 40 nucleotides is optimal for efficient inhibition of HBV entry by NAPs.

## Discussion

Chronic hepatitis B infection remains a major public health problem because current treatments fail to achieve a functional cure. The first indication of NAPs potential against HBV was reported in the DHBV model, demonstrating that NAPs could both prevent infection of ducks and lead to DHBsAg loss and seroconversion in infected animals [17,18]. Furthermore, these studies, along with *in vitro* experiments, demonstrated that NAPs inhibit both viral entry and the release of DHBsAg from cells already infected but that the entry-inhibitory properties of NAPs did not appear as mandatory to their antiviral effects *in vivo* [17,20]. Importantly, the therapeutic effect of REP 2055 and REP 2139 have recently been confirmed in humans in two proof-of-concept clinical trials [19].

Although phylogenetically closely related, Avihepadnaviruses and Orthohepadnaviruses differ in the nature of host cell receptors [27,28] and might require different trans-acting cellular factors to regulate their life cycles [29]. For this reason, we investigated the antiviral activity of NAPs against HBV in HepaRG cells and primary human hepatocyte cultures. We here provide evidence of an inhibitory effect at viral entry. Similar entry antiviral effects in the DHBV and HBV models suggest that NAPs can block an attachment or entry mechanism common to both viral species.

The antiviral activity of NAPs was phosphorothioation-dependent, sequence-independent and size-dependent as previously observed with other viruses [11–14,16] and with DHBV [20]. This structure/activity relationship suggests that the amphipathic nature of NAPs is the major determinant of antiviral activity. However, the reduced inhibition effect of 2’O- methylated NAPs appears unique to HBV and not observed with other viruses [11–15]. However it was not tested with DHBV [20]. Therefore, the target interface for NAP inhibition of HBV entry may have some physiochemical features unique to HBV. Phosphorothioated oligonucleotides have chemical similarity to heparan sulfate [10,26,30,31] and cell surface HSPG are instrumental in the initial attachment of HBV virions to the target cell [26,32]. While experimental proof will be needed, it is therefore reasonable to speculate that NAPs that are able to block HBV entry may compete with cellular HSPG for a low affinity interaction with HBV envelope proteins, thus preventing attachment to hepatocytes. The reduced entry inhibition observed with NAPs bearing the 2’O-Me modification suggests a steric hindrance at the NAP interface involved in blocking viral entry.

Whether the antiviral activity of NAPs *in vivo* is also linked to an immunostimulatory effect will require further investigation, but it appears unlikely, based on a recent study in which REP 2055 treatment in PHH cultures did not elicit significant induction of various cytokine genes in comparison to TLR-1/2 and TLR-3 agonists [18]. This is consistent with the fact that a similar entry activity is observed for NAPs containing (REP 2006) or devoid of CpG motifs (REP 2055 and REP 2031).

The failure to observe a post-entry activity in HBV infected HepaRG and PHH cultures contrasts with the results of previous studies in the duck model, in which post-entry activity was documented [20]. Importantly, the *in vitro* data presented here show that REP 2055 blocks HBV entry much more efficiently than REP 2139, while the antiviral activity for these two compounds is comparable in proof of concept clinical trials [19] suggesting that a post-entry activity of NAPs is likely contributing to the antiviral effects observed *in vivo* and in patients. The absence of a post-entry antiviral effect with NAPs in our *in vitro* setting may be due to a failure to deliver the NAPs to the proper subcellular compartment. Indeed, in previous studies conducted on the uptake of phosphorothioated antisense oligonucleotides (ASOs) that have general chemical properties similar to those of NAPs, it was shown that ASOs in a naked form, were indeed active against liver targets *in vivo* [33–35], but *in vitro*, a simple addition of ASOs to the media in tissue culture did not elicit relevant antisense activity, most likely due to sequestration in endosomal/lysosomal compartments [34,36]. Moreover, the ability of human hepatocytes in primary cultures to traffic naked ASOs to the active subcellular compartment is lost after only 24–36 h in culture [36]. Further investigation to define an efficient delivery system *in vitro* will be required to assess the full extent NAPs activity post-entry.

Altogether, our study demonstrates the ability of NAPs to block HBV entry into hepatocytes. A NAP structure/activity relationship similar to that documented with other enveloped viruses is observed, with the exception of the 2’O-Me modification having a detrimental effect on antiviral activity against HBV. Results from the present study, along with previous *in vivo* and clinical studies, highlight the potential of NAPs as therapeutic antiviral agents in general, and as anti-HBV drug in particular. Combination of NAPs with currently available therapies may represent a new relevant step toward an HBV cure.

## Supporting information

S1 Fig Toxicity of nucleic acid polymers in HBV infected or not HepaRG cells and primary human hepatocytes. Toxicity of REP 2055 and REP 2138 NAP compounds has been assessed in HBV infected (+ HBV) or not (- HBV) HepaRG cells and in PHH using the neutral red assay by treating cells every two days starting two days post-inoculation with a range of 0.07 μM to 10 μM NAP final concentrations. The solvent of NAP compounds was used as a non-treated condition. Puromycine (PUR) at 5 μg/ml was used as positive control of cell toxicity. Three independent experiments were performed to assess the toxicity of REP 2055 and REP 2138 in infected or non-infected HepaRG cells. The toxicity of these NAPs was assessed independently three times in HBV infected PHH and two times in non-infected PHH. All data are expressed as means ± standard deviation. Statistical analysis was conducted with R software using an ordinary one-way ANOVA with random effect for comparison to non-treated sample; *, p < 0.05; **, p < 0.01; ***, p < 0.001; ****, p < 0.0001.
(TIF)
