# Supplementary figures and images for: Inhibition of hepatitis B viral entry by nucleic acid polymers in HepaRG cells and primary human hepatocytes
Source: PLoS One. 2017 Jun 21;12(6):e0179697. doi: 10.1371/journal.pone.0179697 (PMC5479567; doi:10.1371/journal.pone.0179697)

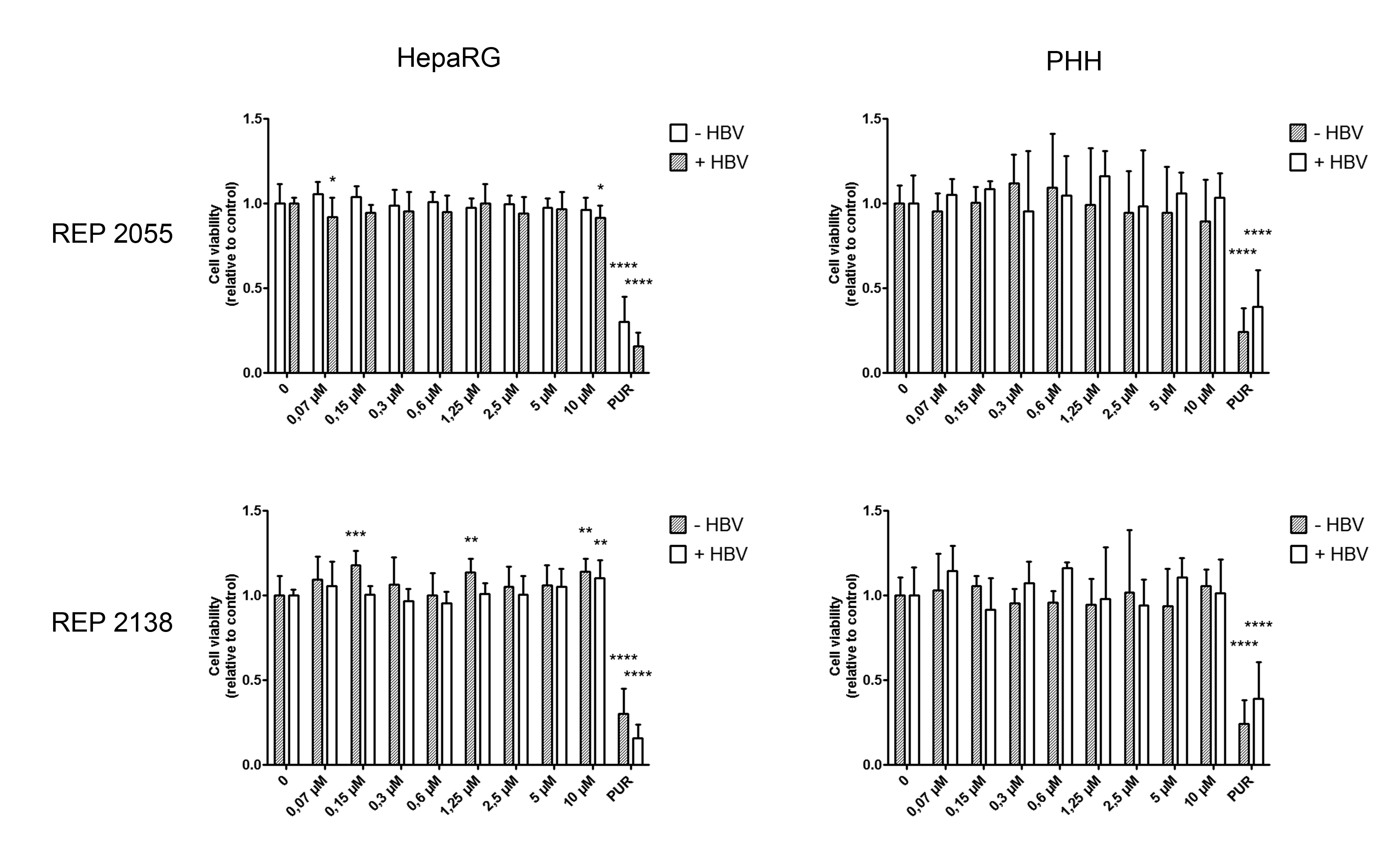

Supplement: S1 Fig — Toxicity of REP 2055 and REP 2138 NAP compounds has been assessed in HBV infected (+ HBV) or not (- HBV) HepaRG cells and in PHH using the neutral red assay by treating cells every two days starting two days post-inoculation with a range of 0.07 μM to 10 μM NAP final concentrations. The solvent of NAP compounds was used as a non-treated condition. Puromycine (PUR) at 5 μg/ml was used as positive control of cell toxicity. Three independent experiments were performed to assess the toxicity of REP 2055 and REP 2138 in infected or non-infected HepaRG cells. The toxicity of these NAPs was assessed independently three times in HBV infected PHH and two times in non-infected PHH. All data are expressed as means ± standard deviation. Statistical analysis was conducted with R software using an ordinary one-way ANOVA with random effect for comparison to non-treated sample; *, p < 0.05; **, p < 0.01; ***, p < 0.001; ****, p < 0.0001. (TIF) [file pone.0179697.s001.tif]
